# Imbalanced IL10/TGF-β production by regulatory T-lymphocytes in patients with HTLV-1-associated myelopathy/ tropical spastic paraparesis

**DOI:** 10.1186/s12879-024-09494-8

**Published:** 2024-06-28

**Authors:** Luana Leandro Gois, Bárbara Ribeiro-Soares, Carlos Gustavo Regis-Silva, Dalila L Zanette, Raphaella Lisboa, Regina Santos Nascimento, Raimundo Coutinho Junior, Bernardo Galvão-Castro, Maria Fernanda Rios Grassi

**Affiliations:** 1 Escola Bahiana de Medicina e Saúde Pública (EBMSP), Salvador-Bahia, Brazil; 2 Laboratório Avançado de Saúde Pública, Instituto Gonçalo Moniz, Fundação Oswaldo Cruz (LASP, IGM, FIOCRUZ), Salvador-Bahia, Brazil; 3 Departamento de Ciências da Biointeração, Instituto de Ciências da Saúde, Universidade Federal da Bahia (ICS/UFBA), Salvador-Bahia, Brazil; 4 Laboratório de Ciências e Tecnologias Aplicadas a Saúde, Instituto Carlos Chagas, Fundação Oswaldo Cruz (ICC/FIOCRUZ-PR), Curitiba-Paraná, Brazil

**Keywords:** HTLV-1, Regulatory T lymphocytes, IL10, TGF-β, HTLV-associated myelopathy

## Abstract

**Background:**

Human T-cell lymphotropic virus type 1 (HTLV-1), also denominated Human T-cell leukemia virus-1, induces immune activation and secretion of proinflammatory cytokines, especially in individuals with HTLV-1-associated myelopathy/tropical spastic paraparesis (HAM/TSP). Regulatory T lymphocytes (Tregs) may control of inflammation through the production of regulatory cytokines, including IL10 and TGF-β. In this study we determined the frequencies of CD4 + and CD8 + Tregs in a HAM/TSP population, compared to asymptomatic carriers and uninfected individuals, as well as investigated the profiles of regulatory and inflammatory cytokines.

**Methods:**

Asymptomatic HTLV-1 carriers and HAM/TSP patients were matched by sex and age. The frequencies of IL10- and/or TGF-β-producing Tregs were quantified by flow cytometry. Real-time reverse transcription polymerase chain reaction (RT-PCR) was used to quantify HTLV-1 proviral load and the mRNA expression of cytokines and cellular receptors in peripheral blood mononuclear cells.

**Results:**

Total frequencies of CD4 + Tregs, as well as the IL10-producing CD4 + and CD8 + Treg subsets, were statistically higher in patients with HAM/TSP compared to asymptomatic HTLV-1-infected individuals. In addition, a positive correlation was found between the frequency of CD4 + IL10 + Tregs and proviral load in the HAM/TSP patients evaluated. A positive correlation was also observed between gene expression of proinflammatory versus regulatory cytokines only in HAM / TSP group.

**Conclusions:**

A higher frequencies of IL10-producing Tregs were identified in patients with HAM/TSP. Imbalanced production of IL10 in relation to TGF-β may contribute to the increased inflammatory response characteristically seen in HAM/TSP patients.

**Supplementary Information:**

The online version contains supplementary material available at 10.1186/s12879-024-09494-8.

## Background

Worldwide, an estimated 10 to 20 million people are infected with human T-cell lymphotropic virus type 1, also denominated Human T-cell leukemia virus-1(HTLV-1), with Brazil being one of the largest endemic areas for HTLV-1 infection and related diseases [1]. In Bahia, a state in northeastern Brazil, the estimated number of infected individuals is 130,000 [2]. The city of Salvador, the capital of Bahia, is considered the epicenter of infection, with approximately 2% of the population infected [3].

HTLV-1 is the causative agent of adult T-cell leukemia/lymphoma (ATLL), HTLV-1-associated myelopathy/tropical spastic paraparesis (HAM/TSP), uveitis, and infective dermatitis [4–6]. HTLV-1 infection has also been associated with inflammatory manifestations, such as arthritis, bronchiectasis, and keratoconjunctivitis [7–9]. In addition, HTLV-1-infected individuals are more susceptible to infectious diseases, including tuberculosis [10, 11], disseminated strongyloidiasis [12], and Norwegian scabies [13].

Following the infection of host cells, primarily CD4 + T lymphocytes, HTLV-1 integrates into the genome in the form of a provirus, leading to chronic and persistent infection. The HTLV-1 Tax protein, which is essential for viral replication, transactivates cellular genes involved in T lymphocyte proliferation and proinflammatory cytokine production. In addition, the HTLV-1 bZIP factor gene (HBZ) in the pX region encodes protein HBZ, which is also able to induce the proliferation of infected cells and inhibit apoptosis, thereby contributing to cell immortalization [14, 15]. Viral replication occurs primarily by mitotic division [16, 17]. Proviral load, which corresponds to the number of cells harboring the viral genome, is increased in patients with HAM/TSP, ATLL and infective dermatitis, as well as HTLV-1-associated keratoconjunctivitis and/or bronchiectasis, compared to asymptomatic carriers [7, 18–23].

Regulatory T cells (Tregs) are known to participate in the prevention of autoimmunity and controlling immune system hyperactivation. This subset is characterized by the expression of CD25 and Forkhead Box P3transcription factor (FoxP3) and can differentiate from naïve CD4 + and/or CD8 + T lymphocytes in the thymus and periphery [24, 25].

Tregs also play a role in chronic viral infection due to persistent antigens by reducing the magnitude of effector T cell responses, which inhibits immune hyperactivation, and aids in controlling inflammation [26, 27]. Tregs attenuate the activation and effector function of CD4 + and CD8 + T lymphocytes, B cells, natural killer (NK) cells, macrophages and dendritic cells by way of several mechanisms, including the secretion of immunosuppressive cytokines (e.g., IL10 and TGF-β), cell-cell contact, and the inhibition or elimination of antigen-presenting cells [28–30]. IL10 plays a crucial role in controlling immune responses and preventing excessive inflammation caused by pathogens. It achieves this by inhibiting proinflammatory cytokines and chemokines while increasing the expression of their natural antagonists. Moreover, IL10 negatively regulates the expression of co-stimulatory molecules such as CD80, CD86 and MHC class II. TGF-β, on the other hand, exerts an antiproliferative effect on CD4 + T cells by inhibiting IL-2 production and the cell cycle. Additionally, TGF-β inhibits the activity of transcription factors for IFN-γ (T-bet) and IL-4, thereby interfering with the differentiation of Th1 and Th2 cells and reducing the production of proinflammatory cytokines by macrophages [1, 31]. In the context of HTLV-1 infection, the HBZ protein induces the expression of FOXP3 but blocks this transcription factor’s ability to bind to the DNA of infected cells, thereby impairing Treg function [32]. Variable frequencies of Tregs have been reported in HAM/TSP patients, as some studies report a higher frequency [33–35], while others find a reduction in CD4 + Tregs compared to healthy individuals [36, 37]. Moreover, the role played by CD4 + and CD8 + Tregs in the development of HAM/TSP, as well as the profile of regulatory cytokine production in these patients need to be investigated.

The present study sought to evaluate, in a group of HAM/TSP individuals compared with asymptomatic HTLV-1 carriers, a panel of regulatory cytokines produced by CD4 + and CD8 + Treg subsets, in addition to regulatory gene expression and proviral load.

## Methods

### Study population

In this cross-sectional study, a total of 32 HTLV-1-infected individuals (15 HAM/TSP and 17 asymptomatic) from the Integrated and Multidisciplinary HTLV Center (CHTLV) at the Bahiana School of Medicine and Public Health (EBMSP-Bahia, Brazil) were recruited between March 2016 and March 2019 [37, 38]. Inclusion criteria consisted of a diagnosis of HTLV-1 infection confirmed by enzyme-linked immunosorbent assay (ELISA) and Western blot. All patients were evaluated by a neurologist to determine the clinical status of infection. Fifteen individuals were classified as definite HAM/TSP and 17 as asymptomatic HTLV-1-infected carriers in accordance with the De Castro-Costa criteria [39]. In addition, a healthy control group with negative HTLV-1 serology was included, who were recruited among CHTLV workers. Exclusion criteria included coinfection with HIV and/or HCV, and current corticosteroid use.

The study was approved by the Gonçalo Moniz Institute Institutional Review Board (Protocol 207/2009). All procedures were planned and carried out in accordance with the ethical principles established by the Declaration of Helsinki and all study subjects provided informed consent.

### Quantification of CD4 + and CD8 + T regulatory cells

Peripheral blood mononuclear cells (PBMC), obtained by Ficoll-Hypaque density gradient centrifugation (Sigma Chemical Co., St. Louis, MO), were cultured for 24 h at 37 °C under 5% CO2. After 19 h, brefeldin A (4 µg/mL) (Sigma) and monesin (4 µg/mL) (Sigma) were added. Five hours later, cells were stained with anti-CD3-APC-Cy7, anti-CD4-AlexaFluor-700, anti-CD8-PECy7 and anti-CD25-PECy5, or isotype controls (BD Biosciences, San Diego, CA, USA) (Supplementary Fig. 1). Cells were then fixed and permeabilized with 1x Fix/Perm buffer (Biolegend, San Diego, CA, EUA) according to the manufacturer’s instructions. Subsequently, the permeabilized cells were stained with anti-FOXP3- PE (BD Biosciences, San Diego, CA, USA), anti-TGF-β-FITC (Biolegend) and anti-IL-10-APC, or isotype controls (Biolegend). Using a FACS FORTESSA flow cytometer (BD bioscience, San Diego, CA, USA), at least 100,000 events were acquired per sample. Flow cytometric readings were analyzed using FlowJo software (FlowJo LLC, Ashland, OR, USA). Treg subsets were defined as CD4 + CD3 + CD25 + FOXP3 + cells (CD4 + Treg) or CD8 + CD3 + CD25 + FOXP3 + cells (CD8 + Treg). The frequencies of cells expressing IL10+/TGF-b-, IL10+/TGF-b+, IL10-/TGF-b + and IL10-/TGF-b- were determined within both CD4 + and CD8 + Treg subsets.

### HTLV-1 proviral load measurement

HTLV-1 proviral load was quantified using a real-time TaqMan polymerase chain reaction (PCR) method, as described previously [40]. Briefly, SK110/SK111 primers were used to amplify a 186 bp fragment of the pol gene with a dual TaqMan probe (5′-FAM/5′ VIC and 3′-TAMRA) located at 4829–4858 bp of the HTLV-1 reference sequence (HTLVATK). Albumin DNA was used as an endogenous reference. The detection limit was established by the standard curve, corresponding to 101 copies. HTLV-1 proviral load values were calculated as follows: [(HTLV-1 average copy number)/(albumin average copy number)] × 2 × 10^6^, expressed as the number of HTLV-1 copies per 10^6^ cells.

### Expression of inflammatory and regulatory genes

RNA was isolated from PBMCs obtained from HAM/TSP patients, asymptomatic HTLV-1 carriers and healthy individuals to analyze a selected panel of the main immune response genes related to HTLV-1, as determined by findings in the literature. Specific oligonucleotides for the following genes were used: TGF-β1, IL17A, IL10, IL12, IL27, IL6, IL1β, TNF-α, IFN-γ, CXCL9, IL4, CCR4 and IL22. For qPCR reactions, optimization was performed using 0.1 µM of each oligonucleotide with Applied Biosystems’ Power SYBR Master Mix. All reactions were performed in duplicate on a 7500 Real Time PCR System under standard instrument conditions. The 2^-ddCt method was used to calculate relative gene expression, with samples from healthy individuals serving as calibrators. The housekeeping genes HPRT and GAPDH were used to normalize data.

### Statistical analysis

Cell frequency data were expressed as medians and interquartile range. Differences in cell frequency medians among the three groups were evaluated using the Kruskal-Wallis test with Dunn’s posttest. In addition, the proportions of cytokine-producing cells were compared between groups using the chi-square test. Correlations between cell frequency and proviral load, or between gene expression and proviral load, were calculated using Spearman’s correlation coefficient. Differences in gene expression between HAM-TSP individuals and asymptomatic carriers were evaluated using the Mann-Whitney test. Significance was assumed when *p* < 0.05. All data were analyzed using GraphPad Prism software (v5.0, La Jolla, CA, USA).

## Results

A total of 15 HAM/TSP individuals, 17 asymptomatic HTLV-1 carriers and 15 uninfected health controls were included. The percentage of females, mean age and proviral load values are shown in Table 1. The mean age of the asymptomatic group was higher than that of the uninfected control group (*p* = 0.04) (Table 1).

**Table 1:** Characteristics of included patients

Characteristics	HAM/TSP (*n* = 15)	Asymptomatic (*n* = 17)	Uninfected controls (*n* = 15)	*p*-value
Females (%)	60%	70%	60%	
Age (years)	51.3 ± 12.1	56.7 ± 14.1^*^	40.7 ± 12.2	0.04*
Proviral load	48,012 ± 19,089	29,662 ± 23,122	NA	

Age and proviral load presented as mean ± standard deviation (SD). NA = not applicable. *Significant difference as calculated using Kruskal-Wallis test, followed by Dunn’s multiple comparison test

**Fig. 1:**
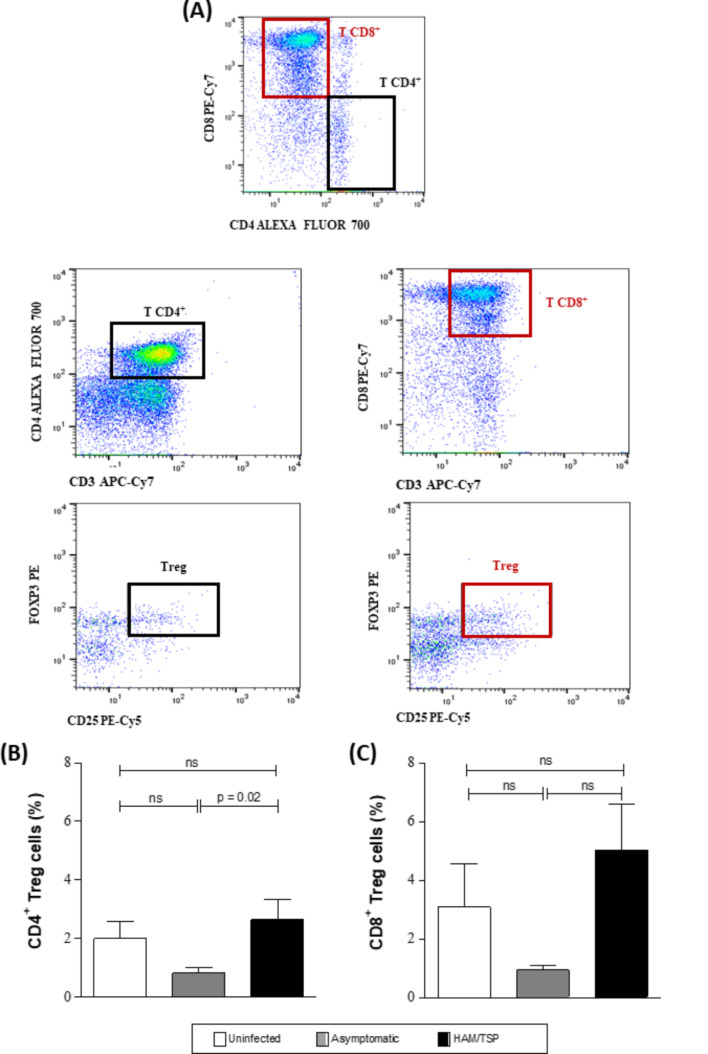
Frequencies of CD4 + and CD8 + Treg cells in HAM/TSP, asymptomatic HTLV-1-infected and uninfected individuals. (**A**) Representative gating strategies to select FoxP3 + and CD25 + cells in CD4 + and CD8 + T cell subsets. (**B**) Frequency of CD4 + Treg cells in HAM/TSP patients (*n* = 15), asymptomatic HTLV-1-infected individuals (*n* = 17) and uninfected controls (*n* = 15). (**C**) Frequencies of CD8 + Treg cells in these same groups. Bars represent median cell frequencies and interquartile ranges. *Significant difference as calculated using the Kruskal-Wallis test, followed by Dunn’s multiple comparison

The frequency of CD4 + Treg cells was found to be 3.4 times higher in the HAM/TSP group compared to asymptomatic individuals (*p* = 0.02) (Fig. 1B), while the frequencies of CD8 + Treg cells were similar among the three groups studied (Fig. 1C).

**Fig. 2:**
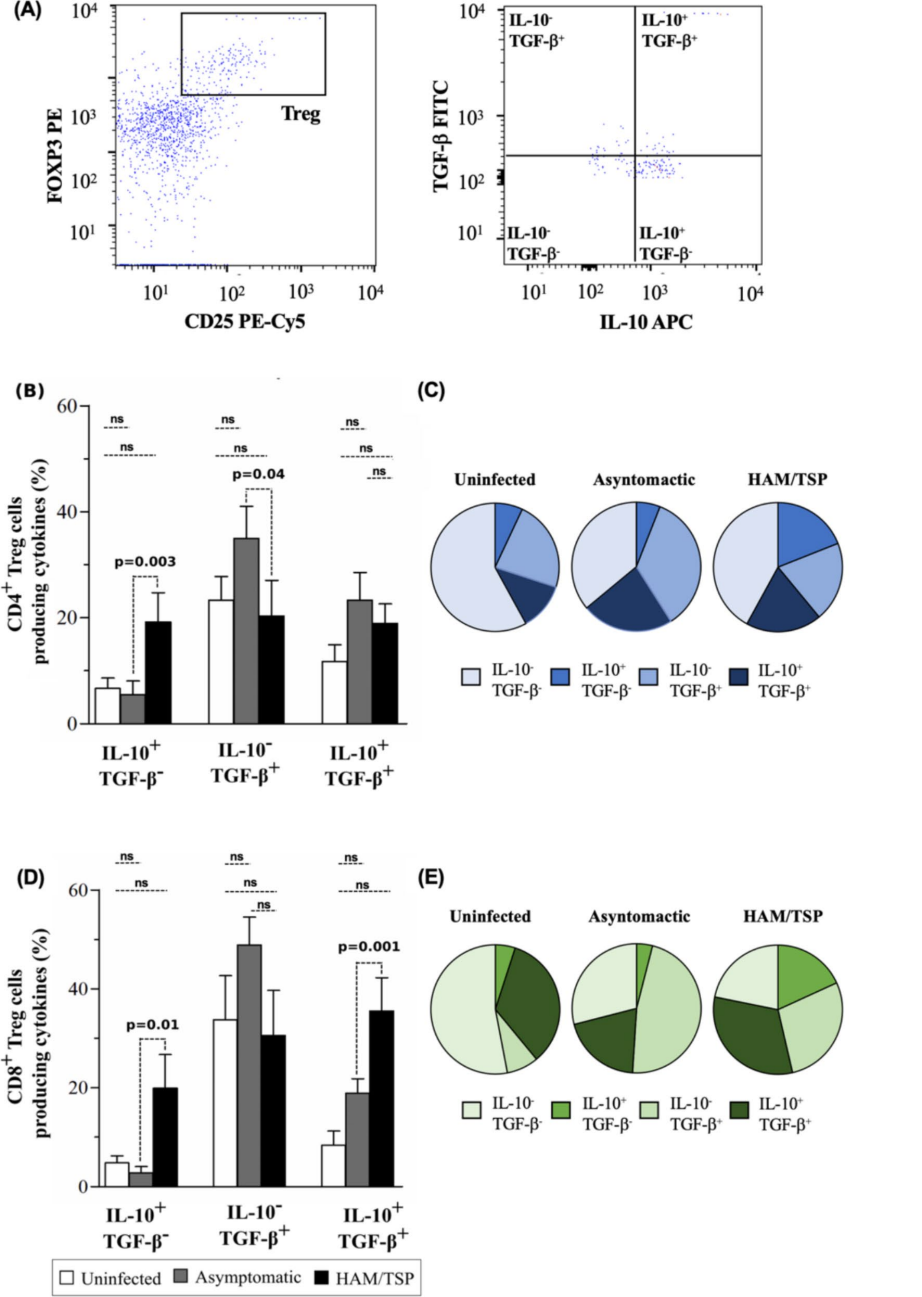
Profile of IL10 and TGF-β production by CD4 + and CD8 + Treg cells in HAM/TSP, HTLV-1-infected and uninfected individuals. (**A**) Representative gating strategy for CD4 + FOXP3 + CD25 + cells (CD4 + Treg) and CD8 + FOXP3 + CD25+ (CD8 + Treg) cells. Frequency of CD4 + Treg cells (**B**) and CD8 + Treg cells (**D**) producing IL10 and/or TGF-β in HAM/TSP patients (*n* = 15), asymptomatic individuals (*n* = 17) and uninfected controls (*n* = 15). Pie charts with the distribution profile of cytokine producing CD4 + and CD8 + Treg cells (**C** and **E**). Data expressed as medians and interquartile ranges. *Significant differences as determined by the Kruskal-Wallis test, followed by Dunn’s multiple comparison post-test

Regarding the frequencies of CD4 + and CD8 + Tregs producing IL-10 and/or TGF-β, a higher frequency of IL10 + TGF-β-CD4 + Tregs was observed only in the HAM/TSP group compared to asymptomatic subjects (*p* = 0.003) (Fig. 2B). By contrast, a lower frequency of IL10-TGF-β + CD4 + Tregs was found only in HAM/TSP patients compared to asymptomatic subjects (*p* = 0.04) (Fig. 2B). Higher frequencies of both IL10 + TGF-β-CD8 + Tregs and IL10 + TGF-β + CD8 + Tregs were observed also in HAM/TSP subjects compared to asymptomatic carriers (*p* = 0.01 and *p* = 0.001, respectively) (Fig. 2D).

Differences were observed with respect to the distribution profile of cytokine-producing CD4 + and CD8 + Treg subsets, with HTLV-1-infected individuals (HAM/TSP and asymptomatic carriers) presenting higher proportions of cells producing IL10 and/or TGF-β compared to uninfected controls (*p* = 0.02 and *p* = 0.0001). In addition, higher proportions of CD4 + and CD8 + Tregs producing only IL-10 were identified in HAM/TSP individuals (19% and 20%, respectively) compared to asymptomatic carriers (6% and 4%, respectively). Conversely, the proportion of CD4 + and CD8 + Tregs exclusively producing TGF-b was found to be higher in asymptomatic carriers (34% and 47%, respectively) compared to HAM/TSP patients (20% and 31%, respectively) (*p* = 0.0009 and *p* = 0.0001) (Fig. 2C and E). As expected, uninfected individuals presented the highest proportion of non-cytokine-producing Tregs compared to either group of HTLV-1-infected individuals (CD4: *p* = 0.02 and CD8: *p* = 0.0001).

**Fig. 3:**
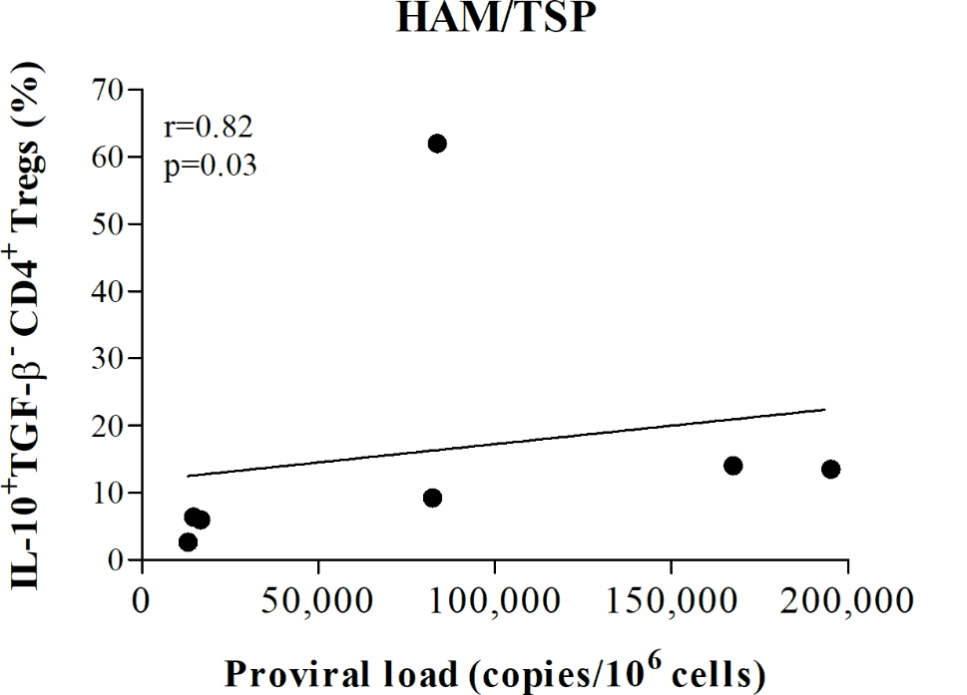
Correlation between proviral load (copies/10^6^ PBMCs) and frequency of IL10 + TGF-b- CD4 + Treg cells in HTLV-1-infected individuals diagnosed with HAM/TSP (*n* = 7) (Spearman’s correlation, *p* < 0.05)

A positive correlation between proviral load and the frequency of IL10-producing CD4 + Treg cells was found only in HAM/TSP patients (*r* = 0.8; *p* = 0.03), as shown in Fig. 3. No significant correlation was observed between proviral load and the frequency of TGF-β-producing CD4 + and CD8 + Treg cells in the HAM/TSP and asymptomatic groups.

**Fig. 4:**
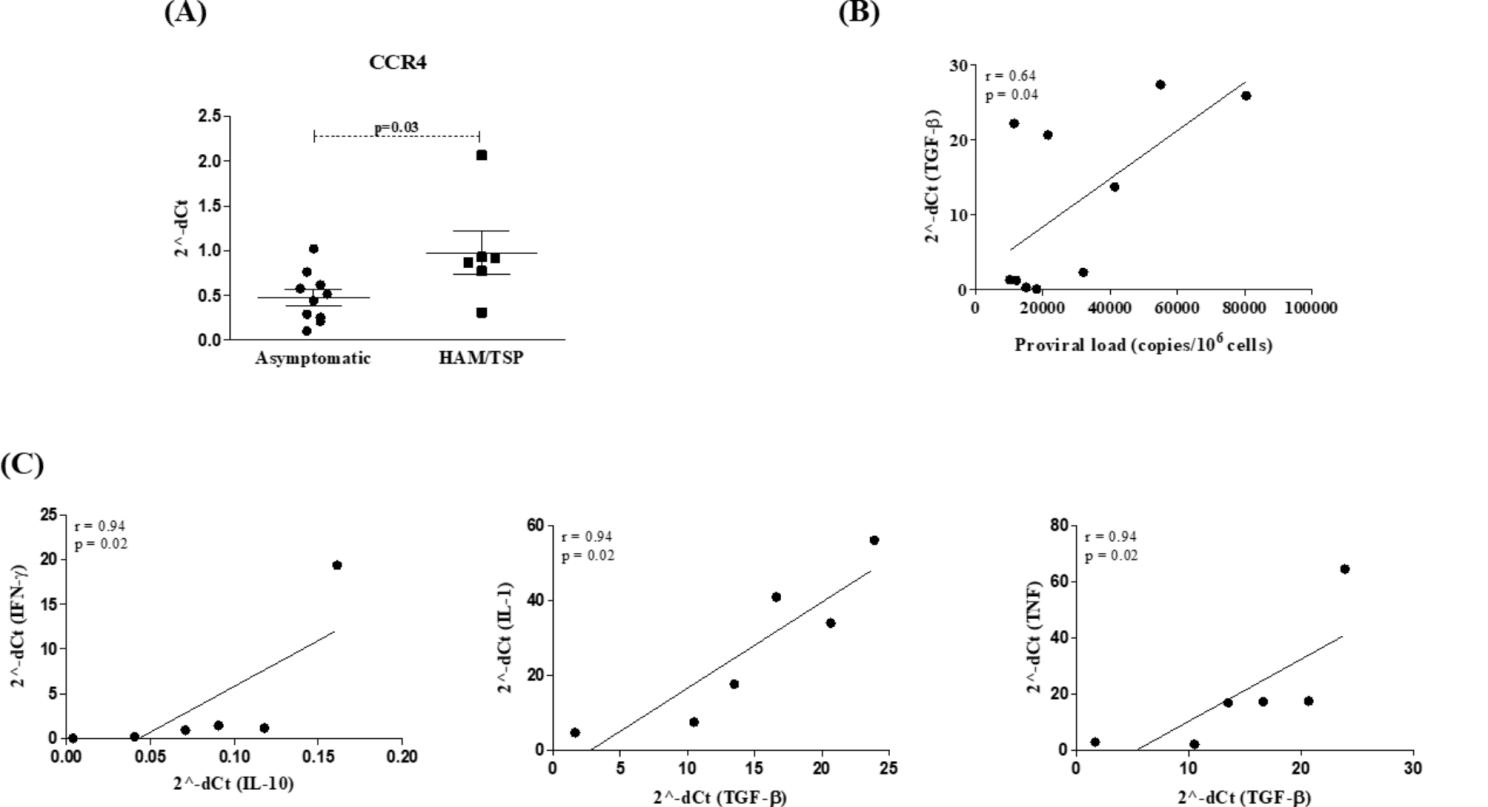
Evaluation of cytokine/chemokine and receptor gene expression in HAM/TSP patients and asymptomatic individuals. (**A**) Gene expression of CCR4 (2^dCt) in HAM/TSP patients (*n* = 6) and asymptomatic individuals (*n* = 10). *Significant differences as determined by the Mann-Whitney test. (**B**) Correlation between proviral load (copies/106 PBMCs) and TFG-b gene expression in asymptomatic HTLV-1-infected individuals. (**C**) Correlation between gene expression of inflammatory cytokines (IFN-g, IL1b and TNF) and regulatory cytokines (IL10 and TGF-b) in HTLV-1-infected individuals diagnosed with HAM/TSP. Spearman’s correlation, *p* < 0.05)

Cytokine/chemokine and CCR4 receptor gene expression was compared between HAM/TSP patients and asymptomatic individuals, using healthy controls as a calibrator. Only the expression of CCR4 was found to be significantly enhanced in HAM/TSP patients compared to asymptomatic individuals (*p* = 0.02). In addition, a significant positive correlation was found between proviral load and TGF-β gene expression exclusively in the group of asymptomatic infected individuals (*r* = 0.64; *p* = 0.04). In the patients with HAM/TSP, significant positive correlations were observed between the expression of IL1β and TGF-β (*r* = 0.94, *p* = 0.02), TNF-α and TGF-β (*r* = 0.94, *p* = 0.02), and also between IFN-γ and IL10 (*r* = 0.94, *p* = 0.02) (Fig. 4). No significant correlation was found between the expression of other cytokine/chemokine genes, the proviral load and the frequency of CD4 and CD8 Treg cells.

## Discussion

The present study aims to test the hypothesis that individuals with HAM/TSP have an imbalance between the inflammatory/regulatory response that may be associated with virus-induced spinal cord damage compared with asymptomatic carriers. We demonstrated that individuals with HAM/TSP present higher frequencies of Treg cells compared to asymptomatic individuals, especially with respect to the CD4 + Treg subset, while asymptomatic individuals had similar levels to individuals not infected with HTLV-1. The literature provides conflicting results regarding the frequency of Treg cells in HTLV-infected individuals. In agreement with our results, some authors report a higher proportion of Treg cells in patients with HAM/TSP [33–35], while others report a decrease in Treg cells in HTLV-1-infected individuals compared to uninfected controls [36, 37]. These discrepancies could be related to the methodology (cell phenotype or even the profile of the patients included). More importantly, our results describe qualitative changes in HAM/TSP patients: both CD4 + and CD8 + Treg subsets of these patients predominantly express more IL10 than TGF-β compared to asymptomatic individuals. It is important to emphasize that most Treg cells from uninfected individuals do not produce cytokines.

HAM/TSP is a chronic neuroinflammatory disease that causes progressive demyelination of the spinal cord. The mechanisms associated with the development of HAM/TSP are multifactorial and have not yet been clearly elucidated. The spontaneous proliferation of infected and uninfected cells, together with increases in proinflammatory cytokines, such as TNF-α, IFN-γ, IL6, and chemokines (CXCL9 and CXCL10), are frequently described in the peripheral blood and CSF of individuals affected by HAM/TSP [41, 42]. Moreover, HTLV-1 proviral load tends to be higher in HAM/TSP patients than in individuals without neurological symptoms or other virus-associated diseases [19].

Given their central role in controlling inflammatory response, imbalances in Treg cell function could contribute to the immunopathogenesis of HAM/TSP [27, 43]. The higher frequency of IL10-producing Treg cells found in patients with HAM/TSP could inhibit the effector function of CD8 + cytotoxic T cells and impair the apoptosis of infected cells, which may contribute to the maintenance of high proviral load. Indeed, an inverse correlation between the frequency of CD4 + Treg cells and the rate of cell lysis mediated by cytotoxic T cells was previously observed in HAM/TSP patients [35]. Importantly, the proliferation of Treg cells in other chronic and persistent viral infections, such as HIV and hepatitis C, has been associated with the suppression of cytotoxic T cell function [44, 45].

It is also possible that CD4 + Treg cells infected with HTLV-1 may constitute a reservoir for the virus, which could also play a role in the higher proviral load seen in HAM/TSP patients. Although HTLV has been hypothesized to trigger upregulation of FOXP3 expression, Treg cells can lose FOXP3 activity and initiate production of IFN-γ, which may contribute to inflammation in HTLV-1-infected patients [46]. However, in the present study, IFN-γ expression was only examined in whole PBMC. It would be important to examine the intracellular production of IFN-γ in addition to IL10 in CD4 + and CD8 + Treg cells to understand whether HTLV-1 is able to induce Treg cells to produce inflammatory cytokines. The present study identified a positive correlation between the frequency of CD4 + IL10 + Treg cells and HTLV-1proviral load only in HAM/TSP group. Other authors previously reported a positive correlation between CD4 + FOXP3 + Treg cells and proviral load [34]. In addition, a positive correlation was observed between the gene expression of inflammatory (IFN-γ, IL1 and TNF) and regulatory (IL10 and TGF-β) cytokines. This correlation may represent the increase in cytokine expression induced by HTLV-1 infection or indicate the activation of a regulatory immune response that can control the inflammation caused by the virus.

The increased expression of CCR4 in PBMCs from the HAM/TSP patients studied herein is consistent with the higher Treg frequencies detected in these individuals, as CD4 + CD25 + CCR4 + T cells were shown to be the main reservoir of HTLV-1 in HAM/TSP patients [47]. Despite higher CCR4 expression, IFN-γ gene expression was found to be similar between HAM/TSP patients and asymptomatic individuals. Although CD8 + T cells are primarily associated with cytotoxic T lymphocyte function and IFN-γ production, the subset of these cells that secrete IL10 are referred to as CD8 + regulatory T cells [25]. While the role of this specific subpopulation requires further investigation in humans, it has been speculated that it may play a role in attenuating the effector response of cytotoxic T lymphocytes.

Conversely, the present study found that asymptomatic HTLV-1 carriers presented higher frequencies of Treg cells expressing only TGF-β. A previous study described reduced levels of the TGF-β receptor II in HAM/TSP patients, which correlated inversely with proviral load [48]. TGF-β is known to exert potent antiproliferative effects on CD4 + T cells by negatively regulating IL2 production, promoting cell-cycle arrest by suppressing the expression of growth-promoting transcription factors and inducing the expression of specific inhibitors of cyclin-dependent kinase [31, 49]. In contrast, IL10 has a predominantly inhibitory effect on the effector function of T lymphocytes, macrophages, and dendritic cells [28, 30]. Thus, it is plausible that Treg cells may regulate inflammatory response in asymptomatic individuals with HTLV-1 through balanced production of TGF-β and IL10, whereas, in HAM/TSP patients, Treg cells tend to produce more IL10 and less TGF-β. This overabundance of IL10 may suppress viral elimination in HAM/TSP patients and inhibit their ability to control inflammatory response. Indeed, an in vitro study demonstrated that the addition of IL10 to PBMCs from HAM/TSP patients did not modulate the spontaneous production of IFN-γ by these cells [50].

The present study has some limitations in terms of sample size, as only a few patients were included in each group and clinical information was limited. It was not possible to investigate the impact of the timing of HTLV-1 infection and the influence of comorbidities on the Treg cells of the included individuals. Furthermore, it was not possible to assess the expression of inflammatory cytokines or viral genes such as HBZ and Tax in the Treg cells. Such an analysis could help to understand how HTLV-1 affects Treg cell function.

## Conclusions

In conclusion, the present study identified a discrepancy in the cytokine-producing profiles of Treg subsets between HAM/TSP patients and asymptomatic HTLV-1 carriers. An imbalance between IL10 and TGF-β production was observed in Treg cells from individuals with HAM/TSP, notably a higher frequency of IL10-producing cells, which may be involved in the pathogenesis of myelopathy in affected individuals. These findings may prove useful for developing novel approaches aimed at predicting the outcome of infection, or in the identification of potential treatment targets. Therefore, it is important to consider IL10 production as a potential biomarker for the development of HAM/TSP as well as TGF-β or IL10/TGF-β balance as a biomarker for protection. These results should be considered in conjunction with PVL and IFN-γ production in further studies. Further studies should be conducted to determine whether the modulation of inflammatory/regulatory response could impact infected individuals’ ability to control proviral load.

### Electronic supplementary material

Below is the link to the electronic supplementary material.

Supplementary Material 1

## Data Availability

No datasets were generated or analysed during the current study.
