# Supplementary material for: Imbalanced IL10/TGF-β production by regulatory T-lymphocytes in patients with HTLV-1-associated myelopathy/ tropical spastic paraparesis
Source: BMC Infect Dis. 2024 Jun 28;24:652. doi: 10.1186/s12879-024-09494-8 (PMC11214226; doi:10.1186/s12879-024-09494-8)
Supplement: Supplementary file 1 — Supplementary Material 1 [file 12879_2024_9494_MOESM1_ESM.docx]

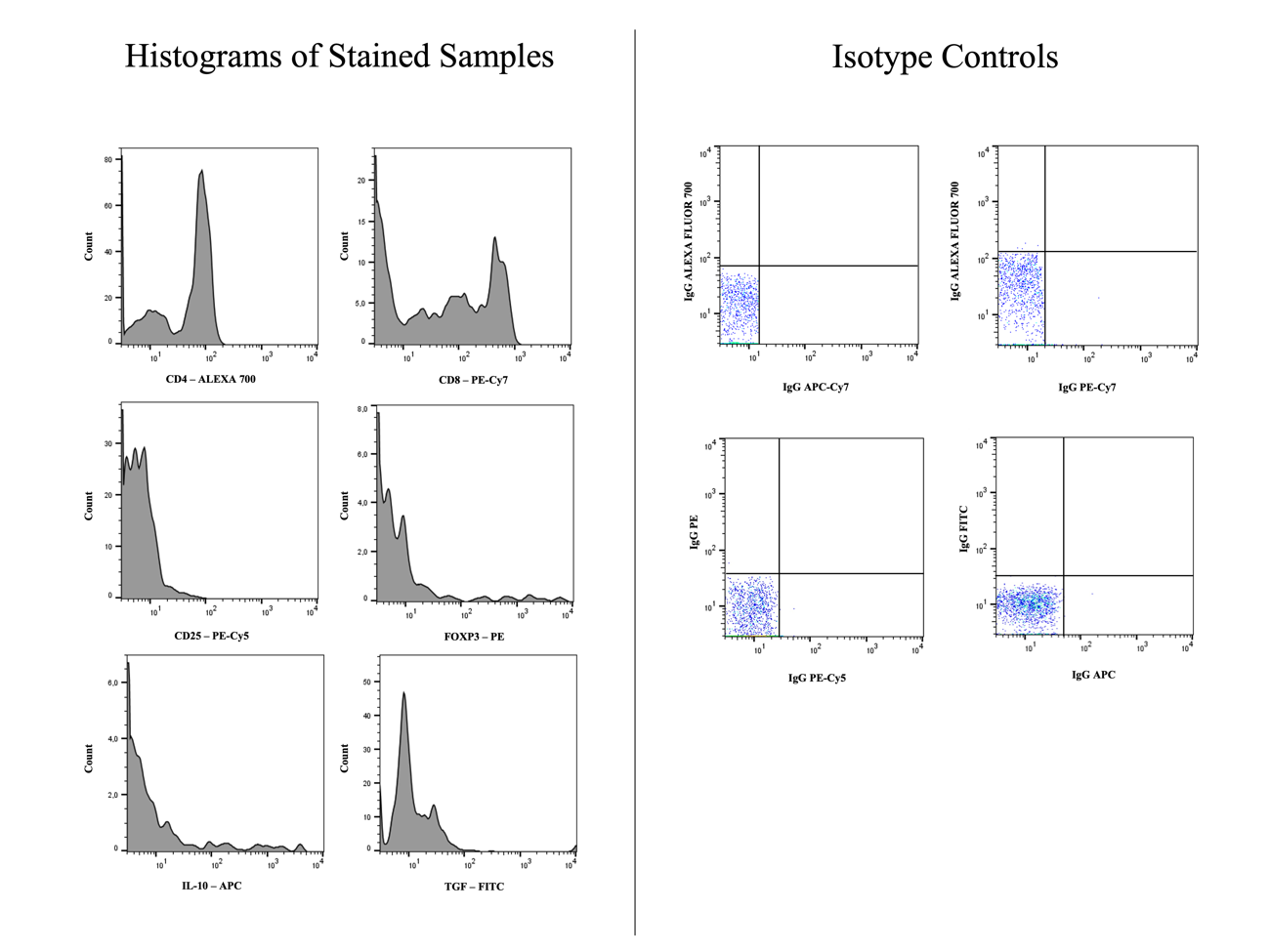


**Supplementary Fig. 1.** **Gating strategies used in flow cytometry analysis of CD4 + and CD8 + regulatory T-cells producing IL10 and/or TGF-β.** A) Histogram of single-marked cells B) Stained cells with isotype controls (IgG2a).
